# Argon Plasma Jet-Treated Poly (Vinyl Alcohol)/Chitosan and PEG 400 Plus *Mangifera indica* Leaf Extract for Electrospun Nanofiber Membranes: In Vitro Study

**DOI:** 10.3390/polym15112559

**Published:** 2023-06-02

**Authors:** Pongphun Sukum, Winita Punyodom, Somsak Dangtip, Pipath Poramapijitwat, Donraporn Daranarong, Thannaphat Jenvoraphot, Mudtorlep Nisoa, Chakkrapong Kuensaen, Dheerawan Boonyawan

**Affiliations:** 1 Doctor of Philosophy Program in Nanoscience and Nanotechnology (International Program/Interdisciplinary), Faculty of Science, Chiang Mai University, Chiang Mai 50200, Thailand; pipath_p@cmu.ac.th; 2 Center of Excellence in Materials Science and Technology, Chiang Mai University, Chiang Mai 50200, Thailand; 3 Department of Chemistry, Faculty of Science, Chiang Mai University, Chiang Mai 50200, Thailand; 4 Department of Chemistry and Center for Innovation in Chemistry, Faculty of Science, Chiang Mai University, Chiang Mai 50200, Thailand; 5 Thailand Institute of Nuclear Technology (Public Organization), Nakhon Nayok 26120, Thailand; 6 Science and Technology Research Institute, Chiang Mai University, Chiang Mai 50200, Thailand; 7 Bioplastic Production Laboratory for Medical Application, Faculty of Science, Chiang Mai University, Chiang Mai 50200, Thailand; 8 Center of Excellence in Plasma Science and Electromagnetic Waves, Walailak University, Nakhon Si Thammarat 80160, Thailand; 9 Research Unit for Bio-Based Innovation, International College of Digital Innovation, Chiang Mai University, Chiang Mai 50200, Thailand; chakkrapong.k@cmu.ac.th; 10 Plasma and Beam Physics Research Facility, Department of Physics and Materials Science, Faculty of Science, Chiang Mai University, Chiang Mai 50200, Thailand

**Keywords:** atmospheric pressure plasma jet treatment, *Mangifera indica* extraction, electrospun nanofibrous membrane, Argon Plasma Jet, HDFa cells

## Abstract

The wound-healing process can be disrupted at any stage due to various internal and external factors. The inflammatory stage of the process plays a vital role in determining the outcome of the wound. Prolonged inflammation due to bacterial infection can lead to tissue damage, slow healing, and complications. Wound dressings made using materials such as poly (vinyl alcohol) (PVA), chitosan (CS), and poly (ethylene glycol) (PEG) with Mangifera extract (ME) added can help reduce infection and inflammation, creating a conducive environment for faster healing. However, creating the electrospun membrane is challenging due to balancing various forces such as rheological behavior, conductivity, and surface tension. To improve the electrospinnability of the polymer solution, an atmospheric pressure plasma jet can induce chemistry in the solution and increase the polarity of the solvent. Thus, this research aims to investigate the effect of plasma treatment on PVA, CS, and PEG polymer solutions and fabricate ME wound dressing via electrospinning. The results indicated that increasing plasma treatment time increased the viscosity of the polymer solution, from 269 mPa∙to 331 mPa∙s after 60 min, and led to an increase in conductivity from 298 mS/cm to 330 mS/cm and an increase in nanofiber diameter from 90 ± 40 nm to 109 ± 49 nm. Incorporating 1% mangiferin extract into an electrospun nanofiber membrane has been found to increase the inhibition rates of *Escherichia coli* and *Staphylococcus aureus* by 29.2% and 61.2%, respectively. Additionally, the fiber diameter decreases when compared with the electrospun nanofiber membrane without ME. Our findings demonstrate that electrospun nanofiber membrane with ME has anti-infective properties and can promote faster wound healing.

## 1. Introduction

The tissue engineering scaffold (TES) is a temporary structure that enables cell and tissue growth, which helps in wound healing. The TES preparation must meet a set of specific requirements, such as specific physical, chemical, and biological properties, which is a challenging task. The production of TES now follows trends that use synthetic, biodegradable polymers. Poly (vinyl alcohol) (PVA), chitosan (CS), and poly (ethylene glycol) 400 (PEG 400) are used in polymers in the biomedical field because they are non-toxic, water-soluble, and biocompatible synthetic polymers. PVA is a water-soluble and biodegradable polymer with high biocompatibility. Even more intriguing, it can crosslink itself because of the abundance of hydroxyl groups on its side chains. On the other hand, PVA is bioinert and undergoes fast hydrolysis; thus, protein and cell adhesion are limited on the pure material [1]. CS is a natural polysaccharide that initiates fibroblast proliferation, aids in the orderly deposition of collagen, and increases the production of natural hyaluronic acid at the site of the wound. Furthermore, CS is a hemostatic agent, stimulating natural blood clotting and blocking nerve endings to reduce pain [2]. Chitosan contains three essential functional groups: an amino group (NH_2_ at C-2), abundant primary hydroxyl groups (OH at C-6), and secondary hydroxyl groups (OH at C-3). These functional groups can easily generate intermolecular hydrogen bonds without disrupting its polymerization and allow for the modification of chitosan chain copolymerization crosslinked with other polymeric chains, allowing for the fabrication of different kinds of composite scaffolds and making it an attractive candidate for tissue repair and regeneration [3]. PVA enhanced the mechanical properties by forming intermolecular and intramolecular hydrogen bonds with the side chains of chitosan, which made the electrospinning processes easier. Polyethylene glycol (PEG) is an FDA-approved biomaterial with good water solubility that is biocompatible, protein-resistant, non-immunogenic, and non-toxic. It may also help in wound healing [4]. PEG 400 increases the solubility of chitosan and plasticizers [5]. 

Electrospinning is a common technique for fabricating polymer nanofiber membranes with diameters at the nanoscale to the µm. Furthermore, electrostatic repulsion is used in the electrospinning process to overcome the surface tension of a solution droplet at the needle tip, stretching the droplet to form a jet that evaporates the solution while flying forward to a collector to fabricate nanofiber membranes [6]. Electrospun nanofibers have been regarded as highly promising for wound dressing applications due to intrinsic properties such as high surface area-to-volume ratio, porosity, swelling, and structural similarity with the skin extracellular matrix [7,8,9]. 

Due to the system of electrospinning requiring a balance of controlled forces, preparing an appropriate polymer solution is the most difficult challenge. To make solutions compatible with the electrospinning process, many studies use mixed solvents or additives. As a result, it is critical to develop a simple and feasible method for increasing the electrospinnability of the polymer solution [2,10,11]. Improving the electrospinnability of polymer solutions is achieved by increasing solution conductivity through the use of solvent mixtures or the addition of salts or plasma-treated solutions [12]. Plasma is the fourth state of matter, consisting of charged particles, electrons, photons, and free radicals. The mechanisms of plasma that involve chemical reactions in solution with active species proceed as follows: First, the active species produced by the ATM plasma jet diffuses into the solution. Next, the dynamic species that can penetrate liquid reacts with chemicals in a polymer solution. Finally, this activation induces and changes the properties of the polymer solution. Recently, pre-electrospinning plasma treatment in different polymer solutions improved electrospinnability and helped to produce thinner nanofibers and bead-free nanofibers. An atmospheric-pressure argon plasma jet treated in a pre-electrospinning solution increased viscosity, conductivity, and surface tension [13], and decreased pH values [12]. Moreover, plasma treatment changed the crystalline phase of nanofibers and affected the surface chemistry of the nanofibers, which was achieved mostly through oxidation of the polymeric chain [12,13]. 

Mango (*Mangifera indica* L.) is one of the most popular tropical fruits. It is in the Anacaediaceae family [14]. Several researchers are interested in the pharmacological properties of mango because the phenolic compounds found in the leaves include C-glycosyl xanthone, flavonoids, polyphenols like mangiferin and gallic acid, and caffeic acid. Mango leaves are used as an analgesic drug. They also have anti-inflammatory, antibacterial, antifungal [14], and antioxidant properties [15] and involvement with the immune system [16] that promotes the wound healing process [17]. For example, ethanol extract from leaves of *Mangifera indica* reduces the acetic acid-induced writhing response model, which evaluates anti-inflammatory and analgesic inhibition against six Gram-positive bacteria (such as *Staphylococcus aureus, Streptococcus agalactiae, Bacillus cereus, Bacillus megaterium, Bacillus subtilis* and *Lactobacillus vulgaricus*), two Gram-negative bacteria (such as *Shigella flexneri* and *Shigella sonei*), and antifungal activity against three species (*Aspergillus ustus, Aspergillus niger,* and *Aspergillus ochraceus* [14].The antiproliferative activity of mango flesh and peel extracts correlates with their phenolic and flavonoid contents [17]. In addition, mango peel contains more polyphenols and flavonoids than flesh and exhibits good antioxidant activity [15,17]. 

In this study, PVA, CS, PEG 400, and Mangifera extract nanofibers were prepared via an electrospinning technique, and the polymer solutions for fabricating nanofiber mats for application in wound dressing were improved. The steps in the present study were as follows: First, PVA, CS, PEG 400, and Mangifera extract were mixed to prepare the polymer solution. Second, the polymer solution was treated with plasma. Lastly, the polymer solution was fabricated into the nanofiber mat. Furthermore, several analysis techniques, including Scanning Electron microscopy (SEM), conductivity, Fourier Transform Infrared (FTIR) spectrometer, and optical emission spectroscopy (OES), were used to evaluate the morphology and chemical properties of the nanofiber membranes.

## 2. Materials and Methods

### 2.1. Polymer Solution

#### 2.1.1. Material

Poly (vinyl alcohol (molecular weight = Approx. 1.15000; Hydrolysis = 98–99 mole%)) was purchased from LOBA CHEMIE PVT. LTD, Mumbai-400005, India. Chitosan (CS) has a degree of deacetylation of about 91.5%. Two percent acetic acid (purity > 99.8%) was purchased from RCI Labscan Limited, Bangkok, Thailand. Poly(ethylene glycol) 400 was purchased from Merck KGaA, Darmstadt, Germany.

#### 2.1.2. Preparation of Polymer Solution

The polymer solution was prepared in a 30 mL mixed solvent of PVA 4.5% *w*/*v* (4.5 g of PVA diluted in 100 mL distilled water), CS 0.05% *w*/*v* (0.05 g of CS dissolved with 2% acetic acid), and poly (ethylene glycol) 400 in a weight ratio of 9:1:0.5 by agitating solution with a magnetic stirrer for 24 h at room temperature before plasma treatment. Then, ME was added to the ratio stated in Table 1.

#### 2.1.3. Plasma Treatment

Pre-electrospinning plasma treatment of the prepared PVA/CS/PEG 400 solutions was performed using an ATM plasma jet generated by a high-voltage radio frequency (HV RF) generator operating at 80–800 kHz, developed in-house by the Plasma Technology for Agricultural Applications Research Laboratory of Walailak University in the Nakhon Si Thammarat province of Thailand [18]. The carrier gas in this work was 99.95% pure argon and typically operated at room temperature with 60% humidity. The effect of plasma exposure time from 0 to 60 min was evaluated. The frequency and power of the radio frequency (RF) power supply, the flow rate of argon gas, and the solution volume were set at 330 kHz, 20 watts, 0.5 L/min, and 30 mL, respectively. The distance between the end of the quartz glass tube and the polymer solution was 20 mm. After plasma treatment, the polymer solution was stored in a bottle with a cap at room temperature for 12 h.

#### 2.1.4. Characterization of Prepared Solutions

The conductivity of various solutions was measured using a conductivity meter. Optical emission spectroscopy (OES) was utilized to monitor the excited species in plasma afterglow, which measured the broad spectrum in the range of 200 to 850 nm. The concentration of nitrite in the solution was measured by the Griess method. Hydrogen peroxide (H_2_O_2_) in solution is immediately measured after plasma exposure by using commercially available reagents (Spectroquant^®^, Merck, Darmstadt, Germany) with a UV-vis spectrophotometer (SPECORD^®^ 50 PLUS, Analytik Jena, Jena, Germany). The BROOOKFIELD viscometer (Halipath stand D, AMETEK Brookfield, Massachusetts, USA) was used to measure the viscosity of the polymer solution.

### 2.2. Mangifera Extraction (ME)

#### 2.2.1. Sample Preparation 

Firstly, *Mangifera indica* leaves were washed with tap water to remove dust and soil on the sample surface. Then, the mango leaves were dried in the oven at 60 °C for 4–6 h. The sample was ground into a fine powder using a mortar and pestle. Finally, the samples were extracted in a Soxhlet extractor with 95% ethanol for 8–24 h [14,19,20]. 

#### 2.2.2. Antioxidant Activity [14,19]

Determination of total antioxidant activity in *Mangifera indica* leaf extract was performed by DPPH assay and ABTS assay. Trolox equivalent (TE) per gram of fresh weight was determined. The Trolox solution was a standard solution, and L-ascorbic acid was a reference compound or a positive control for the DPPH and ABTS assays. DPPH and ABTS assays measure absorbance at 515 nm and 734 nm, respectively. The results were presented as the concentration of the test sample required to scavenge 50% of the DPPH radical. DPPH radical scavenging activity was calculated using the following formula:

(1) $$\%Inhibition=\left(A_{0}-\frac{A_{1}}{A_{0}}\right)\times 100$$

where $A_{0}$ and $A_{1}$ represent the absorbance of the control sample and the absorbance of the tested extract solution, respectively. The control samples contained all of the reagents except the extract. The IC50 value is the concentration of sample required to scavenge 50% of the DPPH free radical and was calculated using non-linear regression analysis from the sigmoidal curve of % inhibition versus concentration [19]. 

The Folin–Ciocalteu method was used to determine the amount of total phenol (TP) [21]. Each extract’s dry mass (50 mg) was combined with 0.5 mL of Folin–Ciocalteu reagent and 7.5 mL of deionized water. After waiting 10 min at room temperature, 1.5 mL of 20% (*w*/*v*) sodium carbonate was added. The mixture was heated in a water bath at 40 °C for 20 min before being cooled in an ice bath. Finally, a UV-vis spectrophotometer (SPECORD^®^ 50 PLUS) was used to measure the absorbance at 755 nm. The results were expressed as gallic acid equivalents (GAEs) per fresh weight. The results of all samples were averaged after they were analyzed in triplicate [21]. 

#### 2.2.3. Antimicrobial Activity [14,22]

##### Disc Diffusion Method (DDM); Zone of Inhibition

Cultures of *E. coli* and *S. aureus* were inoculated separately on the solidified agar. On each Petri dish, 100 mg/mL of test extract, 0.8 mg/mL of ampicillin (positive control), and ethanol were dispensed into the wells. The plates were incubated at 37 °C for 24 h. The sensitivity of the test organisms to the extracts was determined by measuring the diameters of the zone of inhibition surrounding the wells [14,22]. 

##### Determination of Minimum Inhibitory Concentration (MIC) 

The MIC values were determined by preparing seven dilutions of the stock extract solution in bacterial broth. Seven test tubes were arranged in a row, and serial dilutions of the crude extracts were carried out with 100 mg/mL as the highest concentration in tube 1. About 2 mL of nutria broth was poured into each test tube. Then, 2 mL of sample extract was poured into tube 2. It was mixed well, and the process was repeated to produce seven dilutions. The lowest concentration that inhibited the growth of microorganisms was the rough value of MIC and was recorded [14,22]. 

##### Determination of Minimal Bactericidal Concentration (MBC)

Cultures of *E. coli* and *S. aureus* were inoculated separately on solidified agar in each Petri dish with 100 µL of bacterial solution. Then, 5 mL of nutrient agar containing the lowest concentration and the second-lowest concentration of sample extract that inhibited the growth of microorganisms was spread onto the plates. The plates were incubated at 37 °C for 24 h. The number of microorganisms that were growing was determined and recorded to determine MBC [14,22]. 

#### 2.2.4. In Vitro Cytotoxic Study

##### Cell Culture of Human Dermal Fibroblast Adult (HDFa)

HDFa cells were maintained in Dulbecco’s modified Eagle medium (DMEM) GlutaMAX (Gibco; Thermofisher Scientific, Runcorn, UK) supplemented with 10% fetal bovine serum (FBS) (HyClone; GE Healthcare Life Sciences, Hatfield, UK) and 100 units/mL penicillin-streptomycin (Life Technologies, Paisley, UK). Cells were cultured until approximately 80% confluence was achieved before undertaking tests [23]. 

##### Scratch wound healing assay

The cells were seeded in 6-well plates and starved overnight to 24 h before the experiment. The cells were scratched by a scratch tool before adding a test sample at 0.5% ME, 0.75% ME, and 1% ME in the culture medium. After a 24 h incubation period, the cell condition was observed and documented. The distance between the control and treatment cells was then calculated using ImageJ 1.53e software, National Institutes of Health, Madison, WI, USA [24]. 

### 2.3. Electrospun Nanofiber Membrane

#### 2.3.1. Electrospinning Process

**Figure 1:**
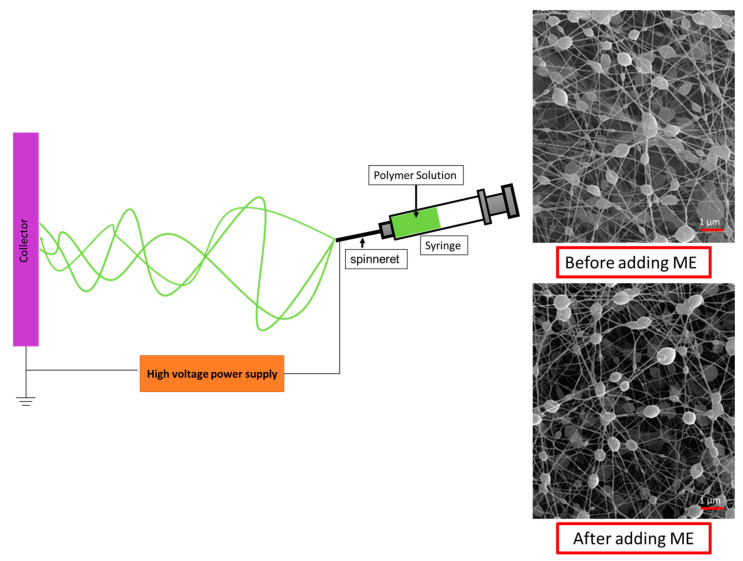
The schematic of the process of electrospinning in a structured and graphic manner.

The mixed solutions were subjected to the electrospinning method. A structured and graphically depicted process of electrospinning is presented in Figure 1. The hybrid solution was loaded into a 5 mL glass syringe equipped with a blunt 22-gauge needle following electrospinning. The syringe tip was connected to the positive output of the high-voltage power supply (20 kV). The distance from the needle tip and collector was 15–20 cm and covered with aluminum foil. The electrospun nanofibers on the surface of the aluminum foil were dried overnight at room temperature [25,26,27,28]. 

#### 2.3.2. Characterization of Nanofiber Membranes

For morphology observation of the nanofiber membranes, the surface of the electrospun samples was coated with a thin layer of gold and scanned by using an SEM analyzing system. A Fourier Transform Infrared (FTIR) spectrometer (NICOLET 6700 FT-IR, Thermo Electron Corporation, Massachusetts, USA) was used to determine the chemical components by identifying the variety of functional groups that explain or highlight their role in membrane performance [29]. The formulation of the polymer solution is shown in Table 1.

#### 2.3.3. Antibacterial Test

This study investigated changes in the antibacterial performance of the electrospun nanofiber membrane with increasing treatment time, using *E*. *coli* and *S*. *aureus* as test organisms. Bacteria were grown on nutrient agar at 37 °C for one day. The bacterial cell count was adjusted to 10^5^ CFU using nutrient agar medium. To test for antibacterial activity, 100 µL of the bacterial suspension was dropped onto four types of electrospun nanofiber membranes: untreated, untreated with 1% ME, plasma-treated, and plasma-treated with 1% ME. The bacterial suspension was then evenly spread using a spreader. The inoculated electrospun nanofiber membrane was dried for 1 h on a clean bench. The dry electrospun nanofiber membrane was immersed in a tube filled with 20 mL of nutrient agar and incubated for 6 h at 37 °C. The cultured suspension was vortexed for 1 min and then used to confirm the antibacterial effect. Survival numbers were obtained by 10-fold serial dilution of bacterial cell suspensions in nutrient agar media, and 100 µL of bacterial suspension was spread on solidified agar plates and incubated for 1 day at 37 °C to obtain viable bacterial counts. The tests were performed on four sample electrospun nanofiber membranes. The antibacterial efficiency or inhibition rate (ƞ) was calculated based on the following equation [30]:

(2) $$ƞ=\frac{\left(N_{B}-N_{A}\right)}{N_{B}}\times 100$$

where $N_{B}$ and $N_{A}$ are the number of viable bacteria control and the number of viable bacteria samples, respectively [30].

The bacteria suspensions were seeded on the plates containing agar, and the nanofiber membrane with a diameter of 6 mm (d) was placed on the plates. The plates were incubated for 24 h. The zones of inhibition (z) of the nanofiber membrane were explored based on the following equation:

(3) $$z=\frac{\left(D-d\right)}{2}$$

where D is the diameter of the outer circle ring.

#### 2.3.4. Scratch Wound Healing Assay [31]

The fabricated nanofibers were covered with fresh sterile medium and were extracted at 37 °C for 24 h. The HDFa cells were seeded in 6-well plates and starved overnight to 24 h before the experiment. The cells were scratched by a scratch tool. The culture medium was then immediately removed and rinsed with PBS (Phosphate Buffered Saline) to clean off any cellular debris. The removed medium was replaced with fresh media (control group) and extract medium (sample). After a 12 h, 24 h, and 48 h incubation period, the cell condition was observed and documented. The distance between the control and treatment cells was then calculated using ImageJ software. We calculated the percentage of wound closure according to [24]:

(4) $$Wound\;area\%\;=\;\left(\frac{A_{t=0}-A_{t=∆t}}{A_{t=0}}\right)\times 100$$

where, $A_{t=0}$ is the initial wound area, $A_{t=∆t}$ is the wound area after n hours of the initial scratch, both in μm^2^.

#### 2.3.5. Statistical Analysis

All the data are presented as mean ± standard deviation (SD) of the mean. The statistical significance between the two means evaluated at *p* ˂ 0.05 was validated using a one-way analysis of variance (ANOVA) and the student’s *t*-test.

## 3. Results and Discussion

### 3.1. Plasma-Treated Polymer Solution

**Figure 2:**
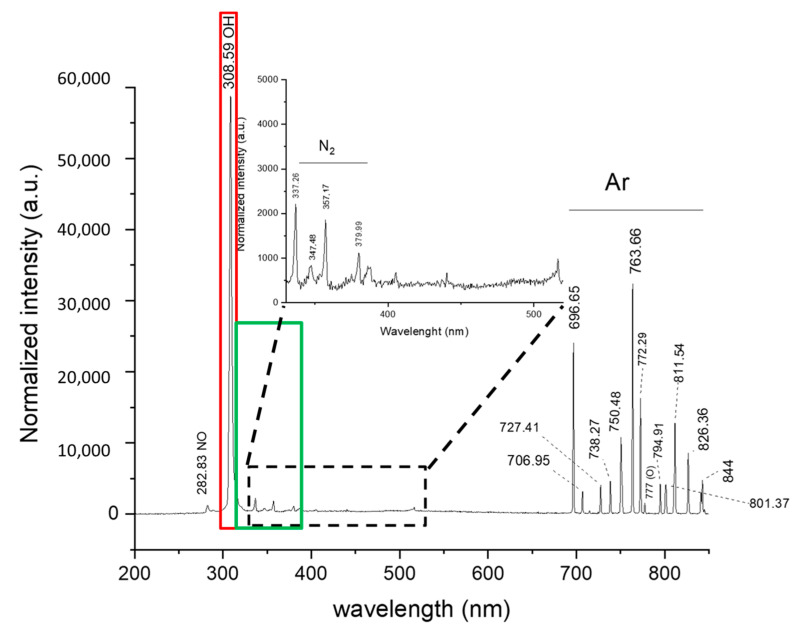
The optical emission spectra of atmospheric-pressure argon plasma jet afterglow in ambient air. (The red line shows the intensity of the OH group, and the green line shows the intensity of N_2_).

This experiment effectively demonstrated the impacts of plasma treatment on a PVA/CS/PEG 400 solution. From the OES results, we were able to identify the chemical species present within the plasma pathway. Specifically, the main exciting active species generated by argon plasma were meticulously analyzed using OES at a broad range of wavelengths, from 200 to 850 nm. According to the emission spectrum results, excited OH radicals, atomic oxygen, and nitrogen were generated in the gas plasma and transferred into the liquid, where they were easily converted to other reactive oxygen species (ROS) and reactive nitrogen species (RNS) in the solution, which included hydrogen peroxide (H_2_O_2_), nitrite (NO_2_^−^), and nitrate (NO_3_^−^). The N_2_ molecule in air undergoes excitation in the plasma phase and emits a photon of characteristic wavelength in the N_2_ second positive system. The observed vibrational mode transitions are at 336.61 nm, 357.17 nm, and 379.99 nm. The weak emission line of the NO radical at 282.83 nm is also observed, which is formed by a reaction between nitrogen and oxygen molecules in the presence of a high-energy third body (electrons, atoms, or molecules). The radiative decay of NO from the upper excited state A2Σ+ → X2Π results in weak emission lines at 282 nm [32]. Moreover, the OH emissions at 308.59 nm were also observable for the argon plasma jet. In Figure 2, strong emission lines of argon were found in the spectral region between 690 and 850 nm, which can be assigned to different excited states of the argon atom. The graphs show intense atomic oxygen at 777.00 and 844.00 nm as humidity components diffused from the surrounding air.

**Figure 3:**
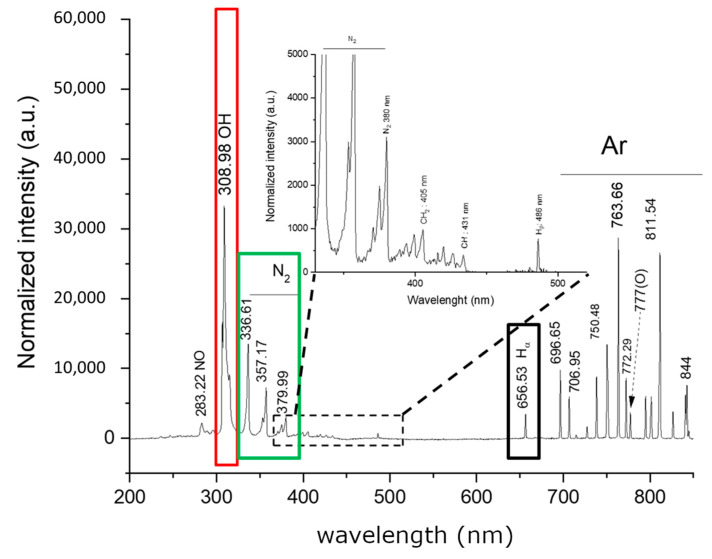
The optical emission spectra of the plasma over the polymer solution. (The red line shows the intensity of the OH group, and the green line shows the intensity of N_2_).

Figure 3 illustrates that when the plasma jet was applied to a polymer solution, the normalized intensity of the argon emission lines increased while the amount of the OH band significantly decreased. The OH signal could be caused by H_2_O impurities in the ambient air and/or on the solution surface. Because of changes in the electron energy distribution, the observed intensity changes in the atomic argon emission lines in the plasma–solution system [12]. There are two significant changes in this spectrum. First, at 405 nm, an additional emission peak attributed to CH_2_ can be observed. Since the H and CH lines were investigated in the OES spectrum of the plasma jet surrounded by solvents alone, these peaks are most likely the result of solution interaction [12]. The presence of excited CH_2_ in the emission spectrum can be attributed to the degradation of the poly(vinyl) alcohol, poly (ethylene glycol), and chitosan polymer chains [12]. Numerous types of gaseous ROS and RNS are generated by surface discharge. Some diffuse across the gas phase before dissolving in liquids. We found that the long-lived species, such as H_2_O_2_, NO_2_^−^, and NO_3_^−^, and the short-lived species, such as ·OH, O_2_, and ·NO, diffused into the solution. 

The purpose of this research is to determine the temperature at PES. Based on the results obtained, the temperature, viscosity, and volume of the plasma-exposed solution (PES) are shown in Table 2. The temperature at the exposure times of 0, 30, and 60 min was 27.1 °C, 27.9 °C, and 29.1 °C, respectively. Our findings are similar to other studies [11], which found that increasing plasma exposure time resulted in increased temperature. The temperature characteristics are caused by the interaction between plasma and PES surfaces [11]. There is a research study on the effect of temperature on PES; it was found that there was a further decrease in the concentration of Reactive Oxygen and Nitrogen Species (RONS) in the water during plasma activation when temperatures were higher than 25 °C [33]. However, from the results, it was found that the temperature of the polymer solution did not differ significantly statistically. Therefore, the amount of ROS did not differ when the treatment time was increased to 60 min. The pH analysis on PES was performed in this study after plasma was exposed to samples for 0 to 60 min. The sample of PES used was a volume of 30 mL and was measured with a digital pH meter. The pH level of PES is shown in Table 2. The pH in PES with plasma exposure for 0, 30, and 60 min was 3.92, 3.98, and 4.02, respectively. The variation in solution pH is also noticeable, and it is thought to be caused by energy transfer between ionized argon species and water molecules, which causes water molecules to dissociate into protons [34]. The increase in pH depends on plasma exposure time. The results indicate that the concentration of hydrogen peroxide and nitrite increased with plasma exposure time. This finding suggests that the concentration of hydrogen ions in the plasma-activated solution drops because of the crosslink reaction via hydrogen bonding between amino groups (-NH_2_) and a hydroxyl group (-OH) of chitosan, and a hydroxyl group (-OH) of PVA [35,36,37]. The conductivity of PES is proportional to the concentrations of charged ions. Furthermore, conductivity is related to pH. The pH value can reflect the concentration of H^+^ in the plasma-activated solution, and the ionization of nitrites and nitrates is the main source of H^+^. Nitrites and nitrates are produced by NO_x_ dissolution, which results from the reactions of N_2_ and O_2_ in the gas phase during discharge, but NO_x_ dissolution tends to decrease as the water temperature increases. As a result, the pH value increases as the temperature increases [33].

The viscosity in PES with plasma exposure for 0, 30, and 60 min was 269.40 mPa∙s, 295.93 mPa∙s, and 330.68 mPa∙s, respectively. The viscosity of the polymer solution increases as the time spent being treated with plasma increases. The viscosity of the solution plays a crucial role in stretching the jet. The plasma-activated solution is known to enhance the conductivity and viscosity of the solution, thereby accelerating the formation of thinner fibers. To produce smooth nanofibers through the electrospinning process, it is advantageous to have a higher conductivity and viscosity. Additionally, using highly viscous polymer solutions prolongs the stress relaxation process, thereby preventing electrospinning jet rupture. Furthermore, polymer solutions with high conductivity reinforce the electric field, leading to improved winding instability and fiber formation [38]. Table 2 depicts the conductivity of the polymer solution. From the results, the conductivity of PES with plasma exposure for 0, 30, and 60 min was 298.33 mS/cm, 318.00 mS/cm, and 330.00 mS/cm. The results demonstrate that increasing the plasma exposure time leads to an increase in the polymer solution’s conductivity. The conductivity of the polymer solution was a variable that could affect the electrospinning process. The high-conductivity polymer solution is superior to the low-conductivity polymer solution in terms of the ability to move electric charge. As a result, the polymer solution with high conductivity has higher tensile strength than the polymer solution with low conductivity, and the fiber produced has a smaller diameter. One possible explanation for these observed changes is the plasma modification causes the solvent molecules to degrade, forming chemical species with high conductivity, such as H_2_O_2_ and/or HNO_3_ [13]. Furthermore, these species are highly polar, resulting in increased polymer solution solubility. This hypothesis is consistent with the previously obtained OES results, showing the solvent molecules degrade during plasma modification [12]. 

Several studies have demonstrated that the conductivity of polymer solutions increases with different plasma exposure duration. Furthermore, the charges generated by the plasma are responsible for the increase in solution conductivity. As a result of charge neutralization, solution conductivities were expected to decrease over time following plasma treatment [2]. Another study showed that solution conductivities decrease over time and that the rate of conductivity decay is affected by solution concentration [11]. The concentration of hydrogen peroxide and nitrite produced by plasma is shown in Table 2. The highest concentration of hydrogen peroxide (H_2_O_2_) was 5.62 ± 0.01 mg/mL with plasma exposure for 60 min, and the highest concentration of nitrite was 15.85 ± 1.01 mg/mL with plasma exposure for 60 min. H_2_O_2_ is primarily produced in PES by the recombination reaction of ·OH radicals generated by plasma at the gas/liquid interface [39,40]. The hydrogen peroxide and nitrite production rate strongly depend on the plasma–liquid interaction at the liquid surface; sputtering and high electric field induce hydrated ion emission and evaporation [41]. This study demonstrates the production of numerous types of gaseous ROS and RNS, such as H_2_O_2_ and NO_2_^−^, through surface discharge. These substances diffuse through the gas phase and eventually dissolve into the liquids. Moreover, when plasma exposure time increases, this leads to a rise in temperature, then the plasma-activated solution evaporates, and hydrogen peroxide and nitrite concentrations are elevated. 

### 3.2. Mangifera Indica Extraction

Wounds are damage to the integrity and continuity of the epithelium caused by a variety of factors. In physiology, wound healing involves repairing the wound. Coagulation, inflammation, proliferation, and remodeling are the four basic phases of this process, provided the appropriate conditions are present to allow them to occur. Polyphenolic compounds (PCs), which include flavonoids and mangiferin, are effective for these therapeutics. These noticeable phytochemicals can scavenge free radicals, activate endothelial cell migration, disrupt cell membranes of microorganisms, and inhibit inflammation and pain [17]. The DPPH radical scavenging assay and the ABTS assay were used to determine this capacity [17]. 

DPPH radical scavenging assay and ABTS assay were applied to evaluate the antioxidant activity of mango leaves, and the results are shown in Table 3. The samples were extracted at various times. Sample 1 was collected from October to December 2021, sample 2 was collected from March to May 2021, and sample 3 was collected from June to August 2021. After conducting the DPPH assay, sample 1 exhibited a total antioxidant activity of 0.93 ± 0.19 mg/mL. The ABTS assay revealed a reading of 0.68 ± 0.01 mg/mL for sample 3, and sample 2 had the highest total antioxidant activity with a value of 3.79 ± 0.09 mg/mL. In the ABTS assay, the samples had a total antioxidant activity of 1.28 ± 0.07, 4.48 ± 0.83, and 1.17 ± 0.01 mg/mL in samples 1, 2, and 3, respectively. The quantity of total phenolic content in samples 1, 2, and 3 was 30.07 ± 0.04, 316.17 ± 18.47, and 39.82 ± 6.71 mg of GAE/1 g of the extract, respectively. Sample 2 had the most antioxidant activity when compared with sample 1 and sample 3. Sample 2 demonstrated higher quantities of DPPH radicals, ABTS activity, and total phenol than samples 1 and 3. Specifically, when compared to samples 1 and 3, sample 2 displayed 4.08 times and 5.57 times more DPPH radicals, respectively. When comparing samples 1 and 3, it was evident that sample 1 had 1.37 times more DPPH radicals than sample 3. Furthermore, sample 2 had 3.5 and 3.83 times more ABTS activity than samples 1 and 3, respectively. Similarly, sample 1 had 1.09 times more ABTS activity than sample 3. However, sample 3 demonstrated higher levels of total phenol compared to sample 1, with a difference of 1.3. Sample 2 exhibited total phenol levels that were 10.51 and 7.94 times higher than samples 1 and 3, respectively. The various months of collection of the samples were related to a chemical in the plant because there are many factors that affect chemicals in plants, such as weather, atmosphere, and humidity [42,43].

All wound healing processes involve reactive oxygen species (ROS). ROS help eliminate pathogenic microorganisms when present in low concentrations. When present in higher concentrations, oxidative stress is produced, resulting in cytotoxicity and cell damage around the wound. As a result, the ability of ME chemical compounds to oxidize ROS is crucial and essential [44]. In addition, it can prevent the injury of cells such as fibroblasts, keratinocytes, and endothelial cells [17,45,46,47,48]. Mangiferin also reduces oxidative stress in wounds by stabilizing free radicals [48]. A lack of care and hygiene in the wounds and bacterial infections are the main factors that alter the inflammatory phase of wound healing [48,49,50,51]. Different microorganisms, such as bacteria, fungi, and antigens, can cause contamination that leads to an infection as the epithelium loses its integrity. Wound infections are mainly caused by four types of bacteria: *S. aureus*, *Streptococcus species*, *E. coli*, and *P. aeruginosa*. These bacterial strains can produce endotoxins that, in turn, raise the levels of pro-inflammatory cytokines, such as IL-1 and TNF-α. If left untreated, the wound may become chronic and cannot be healed [17,48,49,50,51].

*Mangifera indica* leaves have been reported to possess antibacterial activity against *E. coli* and *S. aureus*. In addition, some studies have shown that Mangifera extract has antibacterial activity in Gram-negative and Gram-positive bacteria. Mangifera extract is effective in inhibiting the growth of *S. aureus*, and some studies have shown that if the extract is used at a low concentration, it is bacteriostatic [46]. It was found that the presence of phytoconstituents in the leaf extracts may be responsible for the antibacterial activity of the plant [47]. Some reports have shown that the leaves of Mangifera indica contain alkaloids, anthracenosides, coumarins, flavanones, sugars, tannins, steroids, and saponins. Different classes of compounds could be responsible for antibacterial activity. Some of the components found in the crude extract, such as alkaloids and triterpenoids, have been shown to have antibacterial activity [22]. In our study, Mangifera extract has antibacterial activity on both Gram-positive and Gram-negative bacteria. As shown in Table 4, the antimicrobial properties of ME were found to be mild against Gram-positive bacteria (*Staphylococcus aureus*) and poor against Gram-negative (*Escherichia coli*) bacteria. ME showed a zone of inhibition diameter of 11.3 ± 1.2 mm and 9.7 ± 0.6 mm against Gram-positive and Gram-negative bacteria, respectively. On the other hand, the standard antibiotic ampicillin (4 mg/5 mL) showed a zone of inhibition diameter of 34.3 ± 1.2 mm in Gram-positive bacteria and 24.7 ± 0.6 mm in Gram-negative bacteria. Moreover, the zone of inhibition diameter in Gram-positive bacteria (*S. aureus*) for ME was approximately 1.16 times greater than ethanol and about 3.03 times smaller than ampicillin. The zone of inhibition diameter in Gram-negative bacteria (*E. coli*) for ME was 1.04 times more than ethanol and 2.55 times less than ampicillin. Thus, ME has greater antibacterial activity in Gram-positive and Gram-negative bacteria than ethanol. 

Table 5 shows the minimum inhibitory concentration (MIC) and minimal bactericidal concentration (MBC) of *S. aureus* and *E. coli*. The minimal concentration of Mangifera extract that inhibits *S. aureus* and *E. coli* is 6.25 mg/mL. The minimal bactericidal concentration (MBC) of Mangifera extract that inhibits *S. aureus* and *E. coli* is 12.5 mg/mL. The observed low MIC and MBC values against these bacteria mean that the plant has the potential to treat any ailments associated with these bacterial pathogens effectively.

**Figure 4:**
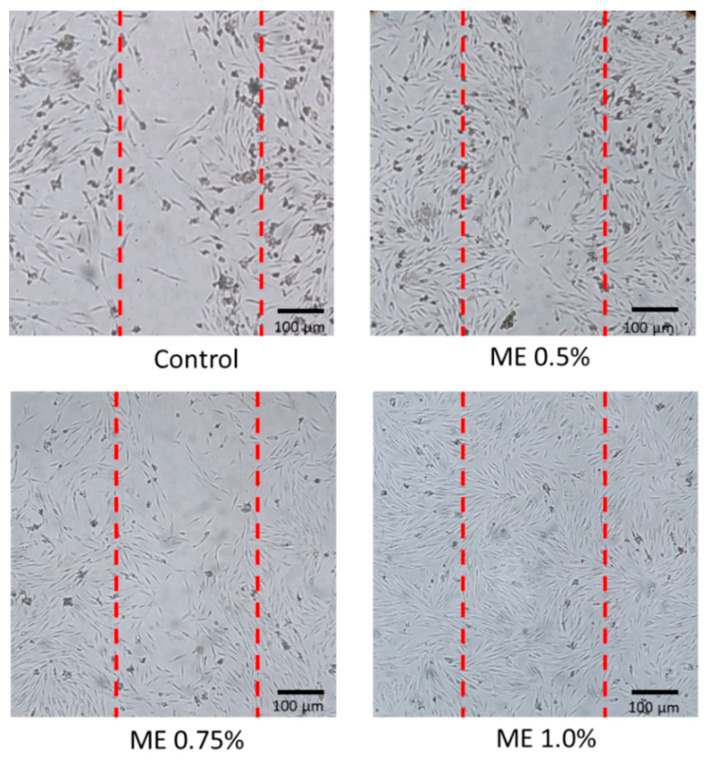
The scratch test was performed on the Mangifera indica extraction after 24 h.

The scratch wound healing assay was carried out to observe the effect of the extract in promoting the migration of cells. The extract’s ability to stimulate cell migration could promote the progression of cells, an important part of wound healing. The results demonstrate that Mangifera extract stimulated cell migration, as seen in Figure 4. The results show that ME 1.0% had the most migration of HDFa cells (ME 1.0% has 0.25% area of wound ±0.24). ME 0.75% (6.18% area of wound ±0.07) and ME 0.5% (6.53% area of wound ±0.06) had less migration than ME 1.0%, and ME 1.0% had more migration than the control group (10.51% area of wound ±0.11). The ME contained a critical substance that was able to allow cells to form cell migration across the cell gap, causing the cells to heal from the gap less than the control group, which shows that the extract may contain important substances that can cause wound healing. The closure of the scratch wound healing assay was incomplete in 24 h [48]. In addition, The HDFa cells in the scratch wound healing assay still stimulated cell proliferation and migration that would complete wound healing in 32–48 h [52]. 

### 3.3. Electrospun Nanofiber Membrane

In this study, it was found that significant increases in the conductivity of PES play a role in improving the electrospinnability of the PVA/CS/PEG 400 solution. As a result, PES was discovered to increase conductivity, which led to more morphologically improved nanofiber membranes. These experiments can aid in finding whether the observed improvement in PVA/CS/PEG 400 nanofiber morphology is due to plasma-induced changes in liquid chemistry, plasma-induced changes in the PVA/CS/PEG 400 polymer chains, or both. 

**Figure 5:**
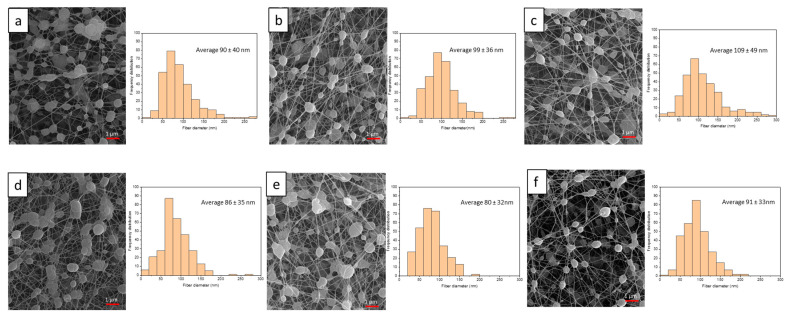
SEM images of 4.5% PVA: 0.05% CS: PEG 400 electrospun nanofibrous membrane without Mangifera extract: (**a**) control, (**b**) plasma exposure time 30 min, and (**c**) plasma exposure time 60 min. SEM images of 4.5% PVA: 0.05% CS: PEG 400 electrospun nanofibrous membrane with 1% Mangifera extract: (**d**) control, (**e**) plasma exposure time 30 min, and (**f**) plasma exposure time 60 min.

The morphology of nanofiber membranes was observed by SEM, as shown in Figure 5. Our results indicate that the significant increase in the conductivity of PES contributes to improving the electrospinnability of the PVA/CS/PEG 400 solution. Furthermore, after PES treatment (for 0, 30, and 60 min, using an argon gas flow rate of 5 L/min and a voltage of 20 kV), the fiber diameter in nanofibrous membranes increased from 90 ± 40 nm to 99 ± 36 nm after 30 min of PES and 109 ± 49 nm after 60 min of PES. Additionally, the number of bead fibers decreased with an increase in the duration of PES treatment. We found that the addition of 1% ME in the polymer solution decreased the fiber diameter, which was 86 ± 35 nm without PES, 80 ± 32 nm after 30 min of PES, and 91 ± 33 nm after 60 min of PES when compared with electrospun nanofiber membranes without 1% ME. These changes in electrospinning paths are most likely the result of the plasma-modified PVA/CS/PEG 400 solution’s significantly increased conductivity [13]. The difference in fiber morphology with voltage is also correlated with changes in the shape of the originating droplet. At low voltages, a solution droplet stays suspended at the end of the syringe needle, and the fiber jet emerges from a cone at the droplet’s bottom. As voltage is increased, the volume of the droplet decreases. The electrospun fibers that are produced retain a cylindrical morphology, but the number of bead defects in the fiber mat increases significantly. When the voltage is increased, the rate of solution removal from the capillary tip exceeds the rate of solution delivery to the tip required to keep the surface conical [12,53]. Other research shows that the electrical conductivity of a solution, in general, reflects a charge density on a jet, thus elongating the level of a jet by an electrical force. As a result, at the same applied voltage and spinning distance, a solution with higher electrical conductivity may cause greater elongation of a jet along its axis, resulting in electrospinning fibers with smaller diameters [37,54,55,56]. However, due to the increased conductivity of the solution after plasma treatment, smooth fibers with fewer beads were formed. As a result of the increased conductivity and viscosity of the solution after plasma treatment, the quality of the nanofibers improved significantly.

Moreover, the viscosity of the solution is important in estimating the fiber diameter and morphology using electrospinning, which measures a material’s resistance to flow. Usually, the solution viscosity is related to the extent of polymer molecule chain entanglement within the solution. Beads are formed instead of fibers at a very low viscosity, and at a very high viscosity, it is difficult to extrude polymer solution; an optimum viscosity is required to form fibers via the electrospinning technique [57,58]. Table 2 shows that as the PES time increases, the viscosity increases and the beads in the nanofiber membrane decrease. The viscosity of the initial solution was 269 mPa∙s, which subsequently increased to 296 mPa∙s after 30 min of plasma treatment. Furthermore, at 60 min, it further increased to 331 mPa∙s, resulting in a significant increase in fiber diameter, from 90 ± 40 nm to 99 ± 36 nm (30 min plasma treatment) and 109 ± 49 nm (60 min plasma treatment). When 1% ME was added to the polymer solution, the fiber diameter was reduced from 86 ± 35 nm to 80 ± 32 nm (30 min plasma treatment) and 91 ± 33 nm (60 min plasma treatment). This is due to the ethanol being nonsolvent in the polymer solution, and the addition of ethanol decreases the surface tension of the solution, resulting in a reduction in viscosity and fiber diameter [59]. 

**Figure 6:**
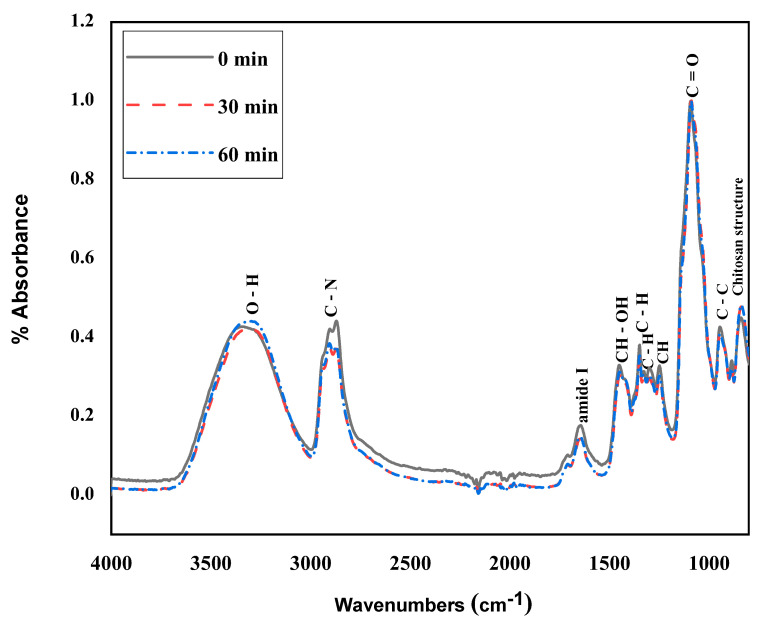
FTIR spectrum of 4.5% PVA: 0.05% CS: PEG 400 electrospun nanofibrous membrane without Mangifera extract from a plasma-exposed polymer solution.

FTIR is a powerful technique for identifying functional groups and material interactions. The FTIR absorption spectra of PVA/CS/PEG400 are shown in Figure 6, which shows a broad absorption band at 3341.3–3314.7 cm^−1^ assigned to O-H stretching vibration [60,61]. At 2904.5–2870 cm^−1^, C-H stretching vibration was observed [62]. The band at 1088.4–1092.1 cm^−1^ indicates the presence of the -C-O group in PVA [63,64]. While the bands at 1643.2–1646.4 cm^−1^ were associated with carbonyl bond (C-O) vibrations of the amide group CONHR (amide I) [65]. The bands at 1247.9–1248.9 cm^−1^ are assigned to the C-H bending vibrations. The band at 1488.7–1448.3 cm^−1^ is an important band that can show many domains for crosslinking in PVA [66,67], as well as a typical band for saccharide structure in CS. The C-C stretching vibration is represented by the bands at 944.7–837.1 cm^−1^ [68,69,70]. In the spectrum of chitosan fibers, a peak was observed at 1348 cm^−1^, attributed to C-N stretching coupled with N-H in-plane strain due to ionic interactions [71]. In addition, with increasing plasma treatment time, the number of C-N bonds decreases. However, the O-H bond content increased with increasing plasma treatment time. The results indicate that the structure of the chitosan fraction interacts with ions, and the polymer interacts with polymer and/or ions (plasma-generated).

Table 6 shows the FTIR spectrum for a nanofibrous membrane with Mangifera extract. The nanofibrous membrane has the absorbance peak intensity at 3322–3341 cm^−1^, assigned to -OH and -NH_2_ stretching vibration. Peak 3408–3309 cm^−1^ is assigned to overlapping -OH and -NH groups. The peak at 1025 cm^−1^ was assigned to skeletal vibration involving C-O of chitosan. The peak observed at 975 cm^−1^ was assigned to amide III of chitosan. Peaks from 920 to 580 cm^−1^ were assigned to pulsation and some types of pyranose ring deformation.

**Figure 7:**
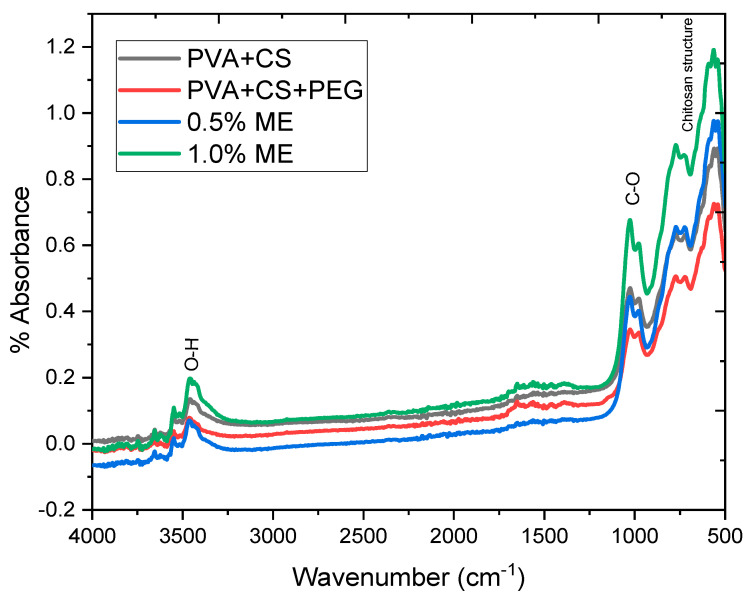
FTIR spectrum of the electrospun nanofibrous membrane that adds Mangifera extract (ME) following Table 1.

Figure 7 shows the FTIR of the electrospun nanofiber membrane followed by Table 1; it offers a broad absorption band at 3100–3500 cm^−1^ assigned to O-H stretching vibration [60,61]. The peak at 1655–1559 cm^−1^ was related to the vibrations of carbonyl bonds of the amide group (CONR). Moreover, the band at 1488–1448 cm^−1^ shows many domains for crosslinking between PVA and CS. The band at 1029–1025 cm^−1^ indicates the presence of the -C-O group [63,64], while the peak at 1025 cm^−1^ is related to stretching vibrations of acetal linkage (C-O-C) [66,67,75]. The bands at 975–830 cm^−^^1^ correspond to C-C stretching vibration [68,69,70,75].

Figure 7 shows intermolecular hydrogen bonds between the hydroxyl group in PVA and the -N-H/C=O bonds in grafted chitosan produced fiber structure in PVA/CS/PEG 400 fiber membranes [72,78,79,80]. Because there was no functional group alternation in the composite fiber, no shifts were detected in the FTIR. The presence of chemical bonds in specific polymers used for composite PVA/CS/PEG400 fiber membranes could be detected using FTIR spectroscopy.

Comparing the Mangifera extract (ME)-loaded PVA/CS/PEG 400 (PCP) electrospun nanofiber membrane and the PCP electrospun nanofiber membrane, the absorbance peak intensity at 3100 to 3500 cm^−^^1^ of the crosslinked PCP electrospun nanofiber membrane reduced when ME was added. This could be due to the hydroxyl and amino group reduction during the crosslinking reaction. According to a previous report [81], the formation of a covalent bond between PVA, CS, and ME crosslinker is a possible crosslinking mechanism for ME because hydroxyl and amino groups react with aldehyde groups via hemiacetal and acetal reactions. The peak attributed to C-O-C stretching vibrations has also been discovered to have shifted to higher-frequency regions because of the formation of bound C-O-C of acetal and ether linkages caused by the reaction between the hydroxyl, amino, and aldehyde groups [82]. The peaks at 1598 cm^−1^ corresponding to NH_2_ were almost eliminated in the crosslinked PVA/CS samples, which can be attributed to the amino group in CS that was consumed due to ME crosslinking. These findings confirmed that the crosslinking reaction between ME and PVA/CS composite nanofibers had occurred successfully. Because of the presence of the crosslinking reaction, the higher the ME concentration, the more hydroxyl and amino groups were consumed, and the more acetal and ether linkages were formed [75,76]. 

**Figure 8:**
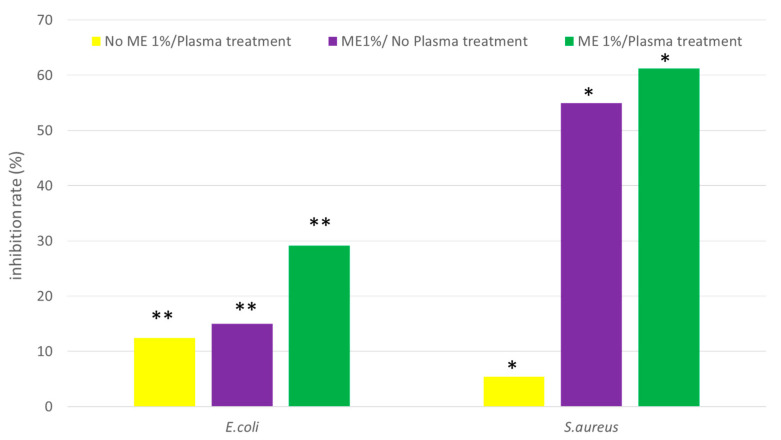
Inhibition rate of electrospun nanofiber membrane; * and ** are *p* < 0.05 and *p* < 0.01.

Figure 8 illustrates the quantities of bacterial colonies present in the electrospun nanofiber membranes subjected to varying treatments, namely the no ME1%/PES nanofiber membrane (NP), no ME1%/PES electrospun nanofiber membrane (EN), and ME1%/PES electrospun nanofiber membrane (EP). Upon comparing these samples to the control group (no ME1%/no PES), it was observed that the inhibition rates of *S. aureus* and *E. coli* were significantly elevated (*p*-value < 0.05 and *p*-value < 0.01). Specifically, the *E. coli* inhibition rates were found to be 12.4%, 14.9%, and 29.2% for NP, EN, and EP, respectively, while the *S. aureus* inhibition rates were 5.4%, 54.9%, and 61.2% for NP, EN, and EP, respectively. Notably, EP demonstrated the greatest extent of bacterial inhibition when compared to the other samples. EP gave the best inhibition rate of *E. coli* at 29.2% and *S. aureus* at 61.2% when compared with the control. 

**Figure 9:**
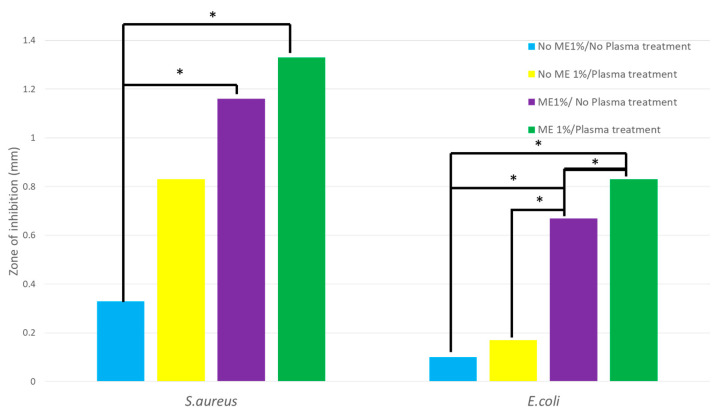
Inhibition zone of electrospun nanofiber membrane; * is *p* < 0.05.

The inhibition zones of the NP, EN, and EP electrospun nanofiber membranes against *E. coli* were 0.2 mm, 0.7 mm, and 0.8 mm, respectively, as seen in Figure 9. The inhibition zones of *E. coli* in different samples were significantly different. The inhibition zones of NP, EN, and EP electrospun nanofiber membranes against *S. aureus* were 0.8 mm, 1.2 mm, and 1.3 mm, respectively. When comparing with the control (no ME1%/no PES electrospun nanofiber membrane and samples (NP, EP)), the inhibition zone of *S. aureus* was significantly increased (*p*-value < 0.05).

Furthermore, the inhibition zones of *E. coli* and *S. aureus* were significantly different between no 1% ME/no PES with EN and EP. The largest inhibitory zone for *S. aureus* was 1.3 mm, while *E. coli* had a zone of 0.8 mm. The results showed that ME1%/Plasma treatment electrospun nanofibrous membrane possessed the best bacterial inhibition ability compared with NN, EN, and NP.

**Figure 10:**
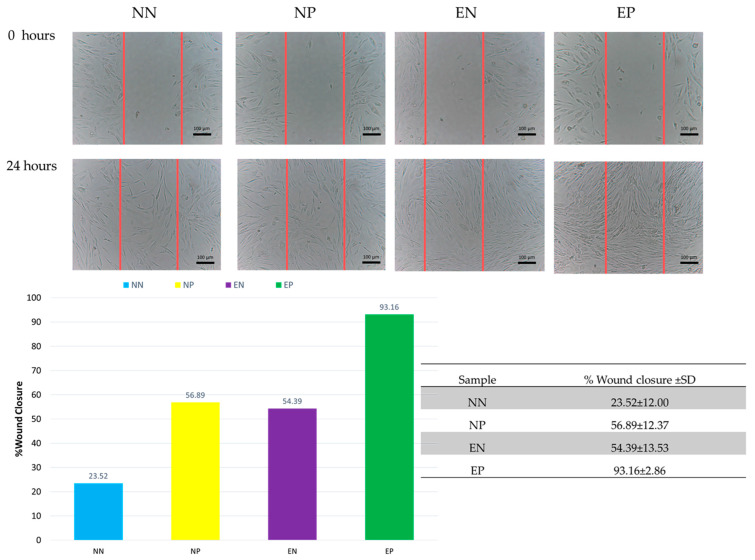
The scratch assay of electrospun nanofiber membrane and % wound closure ±SD.

Figure 10 shows the morphology of HDFa cells treated with ME after 24 h using the scratch assay. This assay was carried out to observe the impact of the electrospun nanofiber membrane on HDFa cell migration. The results suggest that the membrane aided cell migration. After 24 h of treatment, it was observed that the cells had connected, and the gap between them had narrowed. The percent of wound closure for NN, NP, EN, and EP was 23.52 ± 12.00, 56.89 ± 12.37, 54.39 ± 13.53, and 93.16 ± 2.86, respectively. The highest percentage of wound closure was observed in the EP. The capacity of the extract to drive cell migration may improve cell advancement, which is a key aspect of wound healing. The experimental results revealed that the electrospun nanofiber membrane with 1% ME enhanced cell motility. This demonstrated that the electrospun nanofiber membrane with 1% ME had a vital ingredient capable of allowing cells to migrate across the cell gap, indicating that the extract may contain important compounds capable of inducing wound healing. The scratch wound healing assay was completed in 24 h. 

## 4. Conclusions

The study revealed that mango leaves collected from March to May 2021 exhibited greater antioxidant and antimicrobial activity than those collected from October to December 2021 and those collected from June to August 2021. This was due to the difference in seasonal climate, which affected the chemical composition and properties of the samples [62,63]. Argon plasma treatment enhanced the electrospinnability of various polymers, resulting in the production of nanofibers of improved quality. The successful crosslinking reaction between ME and PVA/CS/PEG 400 composite nanofibers was confirmed by the FTIR spectrum. The pH of a polymer solution influenced its conductivity, while various factors, such as solution viscosity and plasma exposure time, affected the morphology of nanofiber membranes. The addition of 1% ME to the polymer solution and the treatment with PES exhibited significant synergistic effects on the electrospun nanofiber membrane’s antibacterial properties, making it a promising option for wound dressing and antibacterial applications.

Overall, the study demonstrates the potential of Mangifera indica extract as an antibacterial agent. Additionally, plasma modification improves the electrospinnability of polymer solutions, resulting in nanofibrous membranes with enhanced antibacterial properties. These findings have significant implications for the development of novel wound dressing materials and other biomedical applications.

## Figures and Tables

**Table 1:** The formulation of the polymer solution, with or without the addition of Mangifera extract (ME).

Sample	Formulation
Control	4.5%PVA/0.05%CS/PEG 400/
0.5% Mangifera extract (0.5% ME)	4.5%PVA0.05%/CS/PEG 400/0.5%Mangifera extract
1.0% Mangifera extract (1.0% ME)	4.5%PVA0.05%/CS/PEG 400/1.0%Mangifera extract

**Table 2:** The characterization of the plasma-activated polymer solution.

Time of Plasma Treatment (Min)	0 Min	30 Min	60 Min
Conductivity (mS/cm)	298.33 ± 1.53	318.00 ± 4.36 *	330.00 ± 6.24 *
Viscosity (cP or mPa∙s)	269.40 ± 1.50	295.93 ± 2.73 *	330.68 ± 2.04 *
Temperature (°C)	27.13 ± 0.21	27.93 ± 0.15	29.07 ± 0.15
pH	3.92 ± 0.01	3.98 ± 0.01	4.01 ± 0.01
NO_2_^−^ (mM)	5.03 ± 3.94	15.79 ± 4.24 *	22.97 ± 1.60 *
NO_2_^−^ (mg/L)	3.47 ± 2.72	10.89 ± 2.93 *	15.85 ± 1.01 *
H_2_O_2_ (mg/L)	0	3.78 ± 0.00	5.62 ± 0.01

* *p* ˂ 0.05.

**Table 3:** The quantity of antioxidant activity in Mangifera extract (ME).

Sample	Total Antioxidant Activity		Total Phenolic Content
	DPPH Assay IC50 (mg/mL)	ABTS Assay IC50 (mg/mL)	Folin–Ciocalteu Reagent (mg GAE/Extract 1 g)
Sample 1	0.93 ± 0.19	1.28 ± 0.07	30.07 ± 0.04
Sample 2	3.79 ± 0.09 *	4.48 ± 0.83 *	316.17 ± 18.47 *
Sample 3	0.68 ± 0.01	1.17 ± 0.01	39.82 ± 6.71

* *p* ˂ 0.05.

**Table 4:** The diameter of anti-bacterial zone inhibition.

Sample	Diameter of Inhibition Zone (Mean ± SD) (mm)	
	*Staphylococcus aureus* (*S. aureus*)	*Escherichia coli* (*E. coli*)
Ethanol	9.7 ± 0.6	9.3 ± 1.2
Mangifera extract	11.3 ± 1.2	9.7 ± 0.6
Ampicillin (4 mg/5 mL)	34.3 ± 1.2	24.7 ± 0.6

**Table 5:** The minimum inhibitory concentration (MIC) and minimal bactericidal concentration (MBC).

Sample	MIC (mg/mL)		MBC (mg/mL)	
	*S. aureus*	*E. coli*	*S. aureus*	*E. coli*
*M. indica*	6.25	6.25	12.5	12.5
Ampicillin	0.8	0.8	0.8	0.8

**Table 6:** Assignment of bands in FTIR for nanofibrous membrane with Mangifera extract [60,62,63,65,72,73,74,75,76,77].

Assignment	Sample			
	PVA + CS	PVA + CS + PEG	0.5% ME	1.0% ME
-OH and -NH_2_ stretching vibration	3322–3341	3100–3500	3452–3652	3452–3652
C-H stretching vibration	2904–2870	2925–2854		
C-O (amide I)	1655–1559	1655–1559	1655–1559	1655–1559
Crosslinking in PVA	1488–1448	1488–1448	1488–1448	1488–1448
C-H bending vibration	1248–1247			
C-O of PVA	1092–1088			
Skeletal vibration involving C-O of chitosan	1029–1025	1029–1025	1029–1025	1029–1025
C-C stretching vibration	944.7–837.1	975–830	975–830	975–830
Pulsation and some types of pyranose ring deformation	920–539	920–539	920–539	920–539

## Data Availability

The data that support the findings of this study are available from the corresponding author upon reasonable request.
